# Retraction: Amorphous selenium nanoparticles improve vascular function in rats with chronic isocarbophos poisoning via inhibiting the apoptosis of vascular endothelial cells

**DOI:** 10.3389/fbioe.2024.1450363

**Published:** 2024-07-01

**Authors:** Frontiers Editorial Office

Following publication, concerns were raised regarding the integrity of the images in the published figures.

The authors failed to provide a satisfactory explanation during the investigation, which was conducted in accordance with Frontiers’ policies. As a result, the data and conclusions of the article have been deemed unreliable and the article has been retracted.

The retraction of the article was approved by the Chief Editor of Frontiers in Bioengineering and Biotechnology and the Chief Executive Editor of Frontiers. The authors did not respond to the retraction.

